# Service user experiences of a nationally implemented diabetes prevention programme in England: a qualitative interview study

**DOI:** 10.1080/21642850.2026.2645889

**Published:** 2026-03-19

**Authors:** Pippa Higgs, Rhiannon E. Hawkes, Lisa M. Miles, Charlotte Dack, David P. French

**Affiliations:** a Department of Psychology, Centre for Motivation and Health Behaviour Change, University of Bath, Bath, UK; b Manchester Centre for Health Psychology, Division of Psychology and Mental Health, School of Health Sciences, Faculty of Biology, Medicine and Health, University of Manchester, Manchester, UK

**Keywords:** Type 2 diabetes, diabetes prevention, behaviour change, service user experiences, interviews

## Abstract

**Introduction:**

Type 2 diabetes (T2D) is a chronic condition linked to obesity that affects millions of people within England. Behaviour change interventions implemented worldwide targeting weight loss have been effective in preventing T2D. In response to this, the NHS Diabetes Prevention Programme (NHS-DPP) was introduced in 2016, and was the first initiative in England to implement a diabetes prevention programme at scale. The current study investigates service users’ experiences of attending in-person sessions of the NHS-DPP when the programme was first rolled out.

**Methods:**

Semi-structured interviews were conducted with service users (*n* = 20) after they completed the programme. Interviews were transcribed verbatim and analysed using a framework approach to thematic analysis.

**Results:**

Three themes were produced. Mindset Shift: whilst the participants did not always maintain their behaviour changes, they experienced a change in mindset towards T2D and their current behaviours. Content tailoring: the participants described how the lack of content tailoring towards culture and the starting weight of participants within the programme negatively impacted their experiences. Social experiences: participants reported that the programme’s group format and wider social support from their friends and family were important for engaging with and maintaining behaviour change.

**Discussion:**

The current study identifies how diabetes prevention programmes can promote behaviour changes and mindset shifts to support diabetes prevention. However, greater content tailoring, particularly concerning culturally informed food preferences and encouraging social support for participants, may improve the long-term effectiveness and engagement of diabetes prevention programmes.

## Introduction

Type 2 diabetes (T2D) is a chronic condition, affecting 6.28% of the global population, which is projected to increase to 639 million diagnoses globally by 2030 (Khan and Hashim, 2020), including 4.9 million individuals in the United Kingdom (UK) by 2035 (Diabetes UK, 2023). T2D is characterised by increased blood glucose levels (HbA1c levels of 48 mmol/mol or higher) due to inadequate insulin production or uptake (Diabetes UK, 2023). Continuous management of blood glucose levels and strict dietary habits are therefore required to maintain glycaemic control and reduce the risk of complications (Berenguera et al., 2016; Gruss et al., 2019). T2D and its associated complications are estimated to cost the National Health Service (NHS) £ 10 billion each year (Ashra et al., 2015), making the prevention of T2D a public health priority (Soares Andrade et al., 2023).

Diabetes prevention trials worldwide have shown that progression to T2D in people at high risk can be slowed or prevented by making changes to health behaviours, including increased physical activity and improved nutrition resulting in weight loss (e.g. Tuomilehto et al., 2001; Knowler et al., 2002). Thus, behavioural interventions have been a focus for T2D prevention interventions at both individual and national scales (Schmidt et al., 2022; Gruss et al., 2019). Following this international evidence, the NHS in England launched the NHS Diabetes Prevention Programme (NHS-DPP) in 2016. This is a nine-month behavioural intervention for adults in England identified as having elevated blood glucose levels and are therefore at increased risk of developing T2D (NHS England, 2021). During the first rollout of the programme, the face-to-face service was delivered by four independent provider organisations commissioned by NHS England through a competitive procurement process (Hawkes et al., 2020a). Exact programme delivery varied between programme providers (see Table 1); however, each provider was required to provide a minimum of 13 in-person group-based sessions lasting 1–2 h over a course of at least nine months (NHS England, 2023) in line with the current evidence base for diabetes prevention programmes (National Institute for Health and Care Excellence, 2017). A digital version of the programme has also been available since 2020 (Moussas-Alvarez et al., 2025), but the current study focuses on the in-person programme.

**Table 1.** NHS-DPP provider programmes.

Provider	Number of sessions	Length of programme	One-to-one support
1	18 sessions 6 weekly sessions, followed by 6 fortnightly sessions, followed by 6 monthly sessions	9 months	No but provided blood glucose test at end of programme
2	13 sessions 8 weekly sessions, and follow-up sessions at 2, 3, 4.5, 6, 9 and 12 months	12 months	Yes – provided during follow up sessions and at end of programme
3	10 sessions (+3 review sessions) 6 weekly group ‘core’ sessions and 4 monthly open group ‘maintenance’ sessions. One-to-one reviews at 3, 6 and 9 months	9 months	Yes – provided during one-to-one reviews and initial assessment at beginning of programme
4	13 sessions 4 weekly closed group ‘core’ sessions, followed by 9 monthly semi-open ‘maintenance’ sessions (people could book onto their own maintenance sessions)	10 months	No but provided blood glucose test (by external provider) at end of programme

Note: Information correct at the time of the first NHS-DPP rollout. Providers are labelled 1, 2, 3 and 4 in this manuscript to preserve anonymity for provider organisations. The labels 1, 2, 3 and 4 do not correspond to labels A, B, C and D used in a previously published article (Miles et al., 2021).

The NHS-DPP represents the first ever diabetes prevention programme to be implemented on a national scale in England (Bower et al., 2023). However, analysis during its early rollout found that only 20% of referred participants completed the NHS-DPP (defined as completing more than 60% of the programme) and consequently were not receiving the intended intervention effects (Marsden et al., 2022). Therefore, there is a need to explore service users’ first-hand experiences with the NHS-DPP to understand where improvements to the service could be made to increase engagement (Ndjaboue et al., 2020). To date, research has captured observations of the in-person NHS-DPP, finding components such as group size and connections with the facilitator and other peers in the group to impact observed participant engagement with the programme (Hawkes et al., 2020b). Similarly, service user experiences of the NHS-DPP pilot programme and digital programme revealed that programme personalisation and support from others were key factors for positive programme engagement (Moussas-Alvarez et al., 2025; Rodrigues et al., 2020). However, no study has directly investigated first-hand service user experiences of the first national rollout of the NHS-DPP and how their experiences might impact on engagement with the face-to-face service.

There is also evidence of considerable variations in programme structure and delivery between providers (Hawkes et al., 2020a; French et al., 2021; Hawkes et al., 2022), including substantial differences in content delivery methods and personalisation between providers (Hawkes et al., 2020b). These differences between providers could significantly impact service user experiences and engagement with the programme. The current study therefore aimed to investigate the experiences of service users who attended the first national rollout of the NHS-DPP delivered via in-person group sessions. The specific objectives were to:

- Explore service user experiences of attending the in-person group sessions of the NHS-DPP
- Explore how service user experiences varied across different provider programmes

## Methods

Methods are reported in line with the Standards for Reporting Qualitative Research (SRQR) checklist (O'Brien et al., 2014). See Appendix A.

### Design

The current study employed qualitative methods, where service users were interviewed about their experiences of attending the in-person service of the NHS-DPP after programme completion (e.g. cross-sectional design).

### Sampling and participants

Participants were recruited from a list of participants (*n* = 101) who had completed the NHS-DPP and consented to be contacted during a previous observation study at eight locations in England where the NHS-DPP was being delivered by four programme providers (Hawkes et al., 2020b). Purposive sampling based on a demographic questionnaire (capturing age, sex, ethnicity, course completion, and site attended) completed by potential participants was used to identify a representative sample (*n* = 32) of the target population representing the eight sites visited during the previous study. Five participants were unable to be contacted. Seven participants withdrew from the study prior to data collection (*n* = 2 due to time restraints, *n* = 1 due to a recent bereavement, *n* = 4 due to an unspecified reason), resulting in a final sample of *n* = 20.

### Procedures

Participants completed one semi-structured telephone interview with a member of the research team (LMM, who was a female Research Associate with training and experience in conducting qualitative interviews) between January and May 2020 after they had completed the NHS-DPP. Full audio-recorded verbal consent was obtained from each participant prior to completing the interview, and socio-demographic characteristics were recorded (age, sex, and ethnicity). Researchers described the aims of the study to participants, which were to understand their experiences and views of attending the NHS-DPP and how their participation in the programme may have helped them make changes to their health behaviours. Each interview lasted between 30 and 60 min. The interviews were audio recorded and transcribed verbatim prior to analysis. Field notes were made following each interview, and recruitment was stopped when the researchers (LMM, REH, and DPF) felt that no new content was being discussed, according to the concept of ‘information power’ (Malterud et al., 2016).

### Materials

The interview schedule comprised questions investigating (1) participants’ exposure to and understanding of behaviour change content delivered in the programme (results of which have been reported separately; Miles et al., 2021) and (2) overall experiences and engagement with the NHS-DPP (reported in this paper). The interview schedule was adapted from a previous interview schedule investigating older patients’ experiences of a walking intervention (Williams et al., 2020).

The initial interview schedule was presented to a patient and public involvement and engagement (PPIE) group varying in age, gender and ethnicity (*n* = 4 people at risk of or living with T2D; *n* = 2 female and *n* = 2 male) for feedback prior to commencing the interviews. The PPIE group provided suggestions regarding expanding questions investigating whether service users’ expectations of the programme were met, the wording of questions, and including a reminder of service users’ right to take a break at any point at the beginning of each interview. This feedback was incorporated into the final interview schedule (see Appendix B for the full interview schedule).

#### Data analysis

The data were analysed using a framework approach to thematic analysis (Gale et al., 2013) to allow comparisons of service user experiences across the four provider programmes. The analysis was aligned with the critical realism approach, which aims to uncover the reasonings behind events and experiences occurring (Lawani, 2021).

Data were analysed by the lead author (PH), who kept an audit trail throughout the analysis. The coding framework was divided into five categories previously identified as important aspects of the NHS-DPP (Hawkes et al., 2020b): ‘General Experiences’, ‘Organisational Experiences’, ‘Educational Experiences’, ‘Support Experiences’, ‘Materials Experiences’ and individual codes were assessed for appropriateness by two researchers (PH, REH). This informed the development of *a priori* codes. The coding framework was piloted on two transcripts and coded using NVivo prior to the full analysis. After the pilot, a primarily deductive approach to analysis was adopted, whereby each transcript was coded against the predetermined framework. However, the framework was adapted throughout the analysis to include inductive codes that were discussed by participants who were not otherwise captured in the framework. This was led by the researcher PH, with regular analysis meetings with a second researcher (REH). For example, the addition of ‘Behaviour Change Maintenance’ was added to the framework to capture the duration of behaviour changes adopted by participants (see Appendix C for the full coding framework). All transcripts were coded using NVivo software. After each transcript was analysed, the data was summarised into a framework matrix by the researcher PH, providing a summary for each participant’s response for each code. The matrix allowed for comparisons to be made across participants and provider programmes for each of the codes, from which themes were developed.

To preserve anonymity, each participant was provided a code using their programme site and their interview number during analysis and a subsequent pseudonym for the final write-up (e.g. KAYLA).

### Researcher positioning

The lead researcher who conducted the analysis (PH) has both a personal background in weight management and has received training in health psychology. This provided the researcher with a strong understanding of behaviour change adoption and maintenance and the ability to identify specific diet culture languages. The researcher who conducted the interviews (LMM) has a background in public health. The wider research team has worked on evaluations of the NHS-DPP for several years (DPF, REH, and LMM). DPF has worked on T2D research projects over the last 20 years, and CD has expertise in clinical and qualitative methods to evaluate and implement public health programmes, including a focus on T2D self-management.

Researchers PH and CD were independent from the wider study team and therefore provided new insights into the data, which were independent from the previous work conducted by the team. The wider team (DPF, REH, and LMM) were able to situate the findings with previous work conducted on the NHS-DPP. This provided the lead author (PH) with greater insight into the geographical sites and provider programmes where participants had been sampled from, contextualising the findings and analysis in the current study.

### Ethical approval

Ethical approval was obtained from the North West Greater Manchester East NHS Research Ethics Committee (Reference: 17/NW/0426, 2^nd^ August 2017). A secondary analysis of the interview data was also reviewed and approved by the University of Bath Psychology Research Ethics Committee (Reference: 4715-5234, 8^th^ August 2024).

## Results

Nineteen of the 20 participants provided demographic data (see Table 2). The median participant age was 65 years (range: 45–80) and there was an even gender split (9m:10f). Seventy percent of the sample classified themselves as White ethnicity. In total, *n* = 4 participants were from provider 1, *n* = 5 from provider 2, *n* = 5 from provider 3, and *n* = 6 from provider 4. The mean course completion rate was 76% (range: 27%–100%), with *n* = 9 achieving 60% completion, and *n* = 6 achieving 100% completion.

**Table 2.** Demographic characteristics of sample interviewed.

Participant (*N* = 20)	Age (Years)	Gender	Ethnicity	Provider	Course completion (%)
Peter	80	Male	White	1	100
Jennifer	68	Female	White	1	94
Larry	62	Male	White	1	94
Lucinda	68	Female	White	1	78
Grace	66	Female	White	2	100
Chris	54	Male	White	2	69
Kellie	67	Female	White	2	62
Anthony	57	Male	White	2	50
Kayla	60	Female	African Caribbean	2	35
Alex	N/A	N/A	N/A	2	N/A
Henry	71	Male	White	3	100
David	66	Male	White	3	100
Nicole	57	Female	Mixed Race	3	92
Caleb	67	Male	Black African	3	67
James	45	Male	White	3	58
Anika	58	Female	Indian	4	100
Violet	72	Female	White	4	100
Kirstie	56	Female	British Asian	4	85
Liam	45	Male	White	4	62
Charlotte	67	Female	White	4	27
**Median**	**66**				**85**
**Range**	**45−80**				**27−100**

Note: ‘N/A’ represents where a participant did not provide demographic data. Providers are labelled 1, 2, 3 and 4 in this manuscript to preserve anonymity for provider organisations. The labels 1, 2, 3 and 4 do not correspond to labels A, B, C, and D used in a previously published article (Miles et al., 2021).

Participants reported similar experiences of the programme across providers. These experiences are reported through three main themes, each with two or more subthemes: Mindset Shift, Content Tailoring, and Social Experiences (see Appendix C for a full breakdown of the coding structure).

### Theme 1 – mindset shift

The participants discussed how their mindset and, subsequently, their behaviours had changed, both in the short and long term, owing to the programme and their recent diagnosis.

#### Mindset change

Despite difficulties in maintaining tangible changes, nearly all the participants showed clear shifts in their mindsets toward diabetes and their health behaviours. For many, being told that they were at high risk of T2D was unexpected, with *n* = 9 participants being diagnosed through a blood test that primarily investigated another factor. Similarly, only *n* = 2 participants expressed a previous understanding of diabetes or prediabetes, suggesting that the initial understanding of diabetes and its risk was low among the cohort. Many participants discussed how their shock diagnosis motivated them to attend the course to gain information and prevent diabetes progression, with some even making changes during the referral process, highlighting an initial mindset shift toward making changes in many participants from the point of diagnosis.

Participants described the course as an ‘​​​​​​*eye-opener’* (ANIKA, 58 years, provider 4), educating them about the risks of T2D and the impact of their current lifestyle on their health. Particularly, the participants highlighted the impact of the initial session that covered how T2D develops, and the health risks of T2D using *‘horror stories’* (LARRY, age 62, provider 1) of individuals with T2D, which acted as a wakeup call for many individuals, priming their mindset towards change going forward. The participants discussed how the course made them question health behaviours such as physical inactivity and portion sizes that they had previously never perceived as problematic and encouraged them to make small but consistent changes throughout.

‘*[The course] made me realise that I needed to change my lifestyle. And to be wary of the things that I never thought could cause you problem end up doing so.’* (CALEB, 67 years, provider 3)

During the interviews, participants displayed a new understanding of the causes and risks of T2D and weight management, accrediting their new understanding to the course, providing clear information and supporting them to navigate the often-complicated world of weight management information. Notably, participants showcased a clear mindset shift towards understanding the impact of individual behaviours on weight management because of the course emphasising overcoming barriers and taking responsibility for individual behaviours, which translated into a clear increased desire to take action to make lifestyle changes to prevent T2D development.

*‘I think the realisation for me was this is something that basically is totally in my hands. I can do something about this… I think the result of that was there were actually no barriers to doing any exercise.’* (KELLIE, 67 years, provider 2)

The participants discussed how their behaviour changes had become part of their daily routines. They mentioned how the course had taught them to take responsibility for their behaviours, including potential relapses, and the importance to *‘just get back on it’* (NICOLE, 57 years, provider 3). Meanwhile, individuals who had reverted to old habits expressed desires to restart their new behaviours, discussing interest in looking over old materials or returning to physical activity once circumstances allowed.

However, despite the clear mindset shift towards taking responsibility for their behaviours amongst participants, many participants expressed desires for further follow-ups after the course finished to maintain their motivation:

*‘One thing is I think it’s good to be, even now, having the odd get together with the tutor or another tutor to make sure you’re doing okay…Well I think at least every six months, if not a bit more but um, I think you can’t just do a course for a year and consider it all okay.’* (ANTHONY, 57 years, provider 2)

#### Behaviour change

All participants reported making behaviour changes due to their course participation. Dietary-based behaviour changes ranged from reduced alcohol and sugar consumption to increased vegetable and protein consumption. Meanwhile, physical activity-based behaviour changes ranged from increased gym attendance to increased daily steps walked, the variation often reflecting individuals’ starting circumstances. The participants discussed making small but regular improvements that progressed to more significant behaviour changes, such as one participant describing how they improved their physical activity levels:

*‘Ten minutes on the bike yesterday, next day I done ten minutes of the exercise bands, next day I’ve done whatever it was, and just upped it the next week fifteen minutes, twenty minutes until I was doing sort of half hour a night.’* (DAVID, 66 years, provider 3)

These changes appeared to be impactful, with all participants reporting weight loss, reduced HbA1c levels, or both, with several participants no longer being classified as prediabetic. The maintenance of these changes was most reported by participants who took part in Provider 3’s programme, who provided one-to-one reviews at months 3, 6 and 9 of the programme (see Table 1), which included tailored goal setting and action planning for each service user. However, participants, particularly those within Provider 2, discussed struggling to maintain their changes over time, despite also receiving one-to-one support. Rather than differences in programme delivery or structure, this may either be due to individual differences across service users, or differences in how the facilitators were delivering aspects of the behaviour change content in the programme. For some service users, problems arose because of overambitious goal setting resulting in failure to achieve goals and subsequent drops in motivation during the course, while others discussed struggling to maintain behaviour changes and weight loss after course completion. Behaviour change relapse was occasionally caused by an uncontrollable factor, such as illness or injury, but many participants felt that their relapse was due to individual resolve. The participants discussed wanes in motivation once they began to see their progress decrease, while others highlighted periods of festivity increasing temptation to relapse.

*‘Over the Christmas period I tasted that piece of chocolate and what my brain was telling me psychologically but it’s Christmas. You can have a bit for Christmas but that’s when I lost the plot so to speak.’* (KELLIE, 67 years, provider 2)

Reactions to relapses varied, with some participants believing that relapses were merely a hurdle to overcome and resume their new habits. However, more often, participants described relapses as an inevitable succumbing to their weaknesses, returning to old habits and subsequently reverting to their starting weight:

*‘I realised after doing the course, not much had altered. I did lose about four kilos and it’s gone back on again.’* (LUCINDA, 68 years, provider 1)

### Theme 2 – content tailoring

The participants raised issues regarding the lack of tailoring of course content for specific needs and circumstances.

#### One size does not fit all

The participants regularly discussed how their cohorts comprised of individuals from a range of backgrounds and ages, requiring tailored interventions to meet their different needs and abilities. The participants acknowledged that there was personalisation in group discussions and problem-solving focused components of the course. Similarly, participants praised the course for the personalisation of the physical activity components of the course, with facilitators providing exercise alternatives to individuals with reduced mobility and highlighting ways to achieve physical activity without attending a gym or running outside.

*‘She showed us how we could do exercise sitting down which I would have never, ever, to be honest, think about doing that.’* (NICOLE, 57 years, provider 3)

Participants described that the programme aimed to prevent T2D through promoting weight loss. However, several participants noted how many members of their cohort were not living with obesity and expressed concerns about whether they would lose more weight. The participants therefore criticised the focus on weight loss, feeling that the group facilitators cared more about the numbers on the scale rather than the individual.

Individual tailoring varied between providers, where participants from Provider 3 reported high levels of tailoring. They received individualised feedback during their one-to-one reviews at months 3, 6 and 9, with their facilitator offering individualised suggestions for potential further changes to those who were struggling to make progress. Conversely, participants from other providers discussed receiving minimal, generic feedback during weigh-ins, with some participants stating that they received nothing beyond a *‘well done’* (PETER, 80 years, provider 1) if their weight had decreased. Notably, Providers 1 and 4 did not offer any one-to-one support as part of their programme, which may explain why some participants perceived their feedback to be generic. In particular, the participants in Provider 2’s programme noted how weigh-ins felt rushed and largely a case of receiving a measurement and returning to their seat, with their limited feedback delivered to the whole group rather than individually.

Overall, the participants across all the providers felt that the course lacked individualisation and acknowledgment of individual backgrounds within the dietary behaviours content, claiming that the programme adopted a *‘one size fits all’* (KAYLA, 60 years, provider 2) approach to content delivery, which negatively affected the experiences of many individuals.

#### Impact of culture

The main factor impacting individuals’ experiences was culture. Every participant in this study who identified as an ethnic minority and some who identified as White, criticised the lack of cultural sensitivity within the programme. While there was no mention of physical activity, participants felt that dietary information on the programme was only appropriate for White individuals. The participants discussed how food swaps suggested by providers ignored the cultural significance of certain foods, such as rice, and the insignificance of other foods, such as bread, claiming there was a lack of awareness of how different cultures perceive foods. There were strong calls for increased acknowledgement of the cultural significance of certain foods within the course to improve individuals’ abilities to engage with the content and make behaviour changes.

*‘If you’re dealing with a client who has diabetes and you’re telling them about eating whole wheat bread, not everybody that eat whole wheat bread, some people eat roti, and like for me I might eat dumplings…it’s not really the best way really to manage a condition like that, because it’s so entrenched in people of diverse, of culturally diverse—that their culture impact on how they eat.’* (KAYLA, 60 years, provider 2)

Elsewhere, participants discussed how many people from ethnic minorities struggled to engage with the programme due to language barriers. One participant (KIRSTIE, 56 years, provider 4) relayed how many members of their cohort spoke English as a second language and struggled with literacy in both their primary language and English, which severely limited their ability to engage with the programme. Moreover, the individuals required many repetitions to try to understand concepts, which potentially reduced the experiences of other cohort members, limiting the facilitator’s ability to cover the required content throughout the session.

*‘Some of the elderly there can’t even read and write their own language, if you know what I mean, but I speak English so it helps me, but I wouldn’t be able to read it or write it. But there was some elderly people there that were their own ethnicity illiterate-wise, like Gujarati, Hindi, Urdu… he managed to get through, but the questions were asked like repetitively.’* (KIRSTIE, 56 years, provider 4)

Another participant (CALEB, 67 years, provider 3) discussed similar experiences, relaying their own limited ability to understand content because of English being their second language, expressing confusion in understanding medical terminology. The participants felt that language barriers prevented some individuals from making and maintaining changes, as they did not understand the material.

#### Content complexity

Most participants felt that the course content was pitched at a low complexity level, covering basic information about weight management, such as food groups and healthy plate composition. Content complexity produced mixed responses among the participants. Some participants believed that a low complexity level was appropriate for the course, feeling many on the course needed to be told the *‘bleeding obvious’* (LARRY, 62 years, provider 1). The participants believed that low content complexity was important to support understanding, often praising the facilitator’s ability to convey the content in simplistic terms when requested and highlighting their improved ease of understanding during the course compared to during conversations with medical professionals.

*‘It was easy to understand, it wasn’t something like my wife will [talk] about a lot of things and she’ll talk about it in GP terms. And I’ll have to say to her, can you explain that to me but a lot of what [health coach name] and the other lady were talking about, everything was easy to understand.’* (LIAM, 45 years, provider 4)

However, many participants expressed dissatisfaction with the low content complexity. The participants wished that the course contained more *‘inspiring’* (LUCINDA, 68 years, provider 1) content through which they could develop their understanding of weight management. These frustrations were particularly common in individuals with previous weight management experience or healthcare backgrounds. Some participants described already knowing most of the content while acknowledging that they still learnt some new information. Whilst others felt that they knew everything covered the course regarding weight management, with some feeling that they knew more than the facilitator delivering the content, which caused frustration among participants. Frustrations with content complexity were most common with Provider 1 participants, who all reported previous weight management experience. This was likely due to individual differences rather than differences between the content of provider programmes. Despite this, most participants felt that while their experience was negatively impacted by the low complexity, the content was pitched correctly to support most individuals attending the programme.

*‘I already did all that but to somebody that came in as a novice, that was brilliant.’* (JENNIFER, 68 years, provider 1)

### Theme 3 – social experiences

Participants described their social experiences during the programme to influence their immediate and long-term experiences, both within their cohort and their wider social circle.

#### Experiences during sessions

Every participant discussed how their cohort influenced their experience of the course. Many participants praised the group format of their course, finding group discussions to be a highlight of their experience. The participants enjoyed interacting with their cohorts, learning about their experiences and feeling safe to express their opinions surrounded by likeminded individuals. Additionally, participants felt that good group discussions could liven up potentially *‘drab’* (JAMES, 45 years, provider 3) topics of conversation through individuals providing different opinions and debate about practical solutions. This was particularly the case within Provider 3 participants, who recalled positive experiences of both practical support from their cohort but also a positive group dynamic consisting of jokes and friendly relationships.

*‘Well to be honest it were enjoyable to go to ‘cos they were a good crowd, and it made it interactive so you knew people were talking about different things. About all I've cooked this and I've had this and everything else saying it.’* (HENRY, 71 years, provider 3)

However, not all participants reported positive group dynamics. The participants, particularly those within Provider 2, discussed struggling to engage with their groups due to characteristics such as age, ethnicity, or work status, meaning that they did not identify with people in their cohort.

The impact of group discussions extended beyond an individual’s enjoyment of each session. Group discussions appeared to be a key element of the course for promoting behaviour change. The participants discussed gaining inspiration to try new strategies or make dietary swaps from hearing the success stories of other participants. Additionally, the participants described using group discussions to identify and overcome potential barriers they faced in making behavioural changes through both facilitator-led discussions and informal discussions between members. The participants mentioned how group discussions provided a safe space through which individuals could discuss struggles and potential relapses and experience support to get back on track with their new behaviours, highlighting how group discussions were important for both promoting and maintaining behaviour change.

*‘I think the group thing was the key to it, because you speak to people and you’re talking about stuff and you think, oh yeah, maybe that’s why I’m not doing this, or maybe yeah, I can do that, you know. If I do that, maybe it might help me as well.’* (ANIKA, 58 years, provider 4)

#### Influences from wider social circles

The participants also discussed the importance of family support in promoting and maintaining behaviour change. While a few participants mentioned not informing anyone of their course participation, most participants aimed to involve their partners and family in their behaviour change process. Many participants relayed positive stories of social support, highlighting how partners had made diet and physical activity changes with them, had driven participants to sessions, and prevented participants from relapsing. Similarly, participants discussed family members, particularly young children, providing verbal encouragement, reminders not to consume sugary foods, and chances to engage in physical activity.

*‘The family were very good. I've got a wife who’s a matriarch so if I tried to touch anything that’s not good for me, I get the stare.’* (HENRY, 71 years, provider 3)

Conversely, some participants found that partners and family members could act as an additional barrier to making behaviour changes. The participants discussed struggling to adapt their meals at home because of their families not being willing to adopt their dietary changes. Some found that their partners and family members would include foods they were aiming to remove when cooking dinners, placing them in uncomfortable situations of having to break their new habits to avoid offending their family.

*‘That was my biggest problem [laughs] because I cook for the family and none of the family—the family wanted proper big meals anyway, so that was my biggest issue, you know, cooking two different meals basically, one for myself and one for them.’* (ANIKA, 58 years, provider 4)

More broadly, certain participants, primarily from White ethnicities, mentioned experiencing dismissal of course participation and rejection of course content from family members. The participants reported that family members may not believe a participant needed to attend the course or what participants relayed to them because of the conflicting information available elsewhere. Dismissal from family members could be disheartening for participants and provided an additional barrier to behaviour change.

Despite some negative experiences, overall social support was reported to be high across all providers. These high levels of social support resulted in an apparent snowball effect, whereby participants’ engagement with the programme also resulted in behaviour change for family members and partners. Many participants’ partners adopted changes to support them, with participants reporting that their partners saw positive results regarding their weight as a result. Additionally, participants discussed passing on their workbooks to partners and family members and that family members had since taken up the programme after being motivated by the participant’s success, highlighting a potential wider impact of the NHS-DPP beyond course members.

## Discussion

Those who attended the in-person group sessions of the NHS-DPP discussed a *Mindset Shift* through which the course increased their understanding of T2D. Their new understanding often translated into behaviour change, although many participants struggled to maintain their changes, particularly once they lost access to their regular follow-ups. The participants also discussed a lack of *Content Tailoring* throughout the programme, which could negatively impact participants’ experiences and subsequent weight loss, particularly for people from ethnic minorities. Participants from different provider programmes showcased different levels of individual tailoring throughout their groups, including both service offer and within-group experiences, particularly around goal setting and individual feedback, which potentially contributed to differences in behaviour change maintenance. Finally, participants discussed how their *Social Experiences* within and outside the sessions impacted on their engagement and behaviour changes both during and after taking part in the programme.

### Comparison with previous literature

Participants often reported being unable to maintain their behaviour changes whilst participating in the NHS-DPP. Previous studies investigating long-term behaviour changes after participation in a diabetes prevention programme found reduced behaviour changes in participants multiple years after programme completion, though participants still displayed significantly more changes than control participants (Lindström et al., 2013; Diabetes Prevention Program Research Group, 2015). Despite difficulties in maintaining behaviour changes, participants expressed clear mindset shifts towards making and maintaining a healthier lifestyle after completing the programme, a factor not previously identified within evaluations of diabetes prevention programmes.

Participants in the current study highlighted social support as a key factor in promoting behaviour change maintenance. Research concerning the effects of social support within the NHS-DPP is inconsistent. One study investigating the digital version of the NHS-DPP reported being less reliant on group support, prioritising intrinsic motivation to engage in behaviour change (Ross et al., 2023), whilst another study found participants reported that support from health coaches and their wider social circle encouraged behaviour change maintenance (Moussas-Alvarez et al., 2025). Hawkes et al. (2020b) reported more positive experiences in NHS-DPP groups with high level of group discussion without disagreements, supporting the findings of the current study that group dynamics could impact programme engagement and behaviour changes. More consistently, previous NHS-DPP research of both in-person (Rodrigues et al., 2020) and digital (Moussas-Alvarez et al., 2025) programmes identified an apparent knock-on effect of individual engagement on the lifestyle behaviours of an individual’s partner or family, in line with the findings of the current study. Potential knock-on effects have also been identified in other diabetes prevention programmes, with previous research identifying both positive and negative knock-on effects of individual engagement within partners (Baucom et al., 2022). Therefore, the findings of the current study support the evidence base highlighting the importance of social support both within the programme from participants as well as wider support from family and friends for promoting behaviour change maintenance.

The participants often discussed how the generalised nature of the programme’s content limited its impact on their behaviour changes. Concerns about a lack of content tailoring within the NHS-DPP were previously raised during service user interviews regarding the NHS-DPP pilot digital programme (Ross et al., 2023). Similarly, an investigation of content provision between providers of the digital version of the NHS-DPP found only one provider included strong content tailoring, with other providers adapting previous in-person programmes to develop their digital offerings (Miles et al., 2023). The participants in the current study frequently highlighted the impact of the lack of cultural adaptations within the programme, despite some ethnic minorities being up to four times more likely to develop T2D than White individuals are (Diabetes UK, 2023), and culturally adapted programmes are more effective in reducing T2D risk in individuals from ethnic minorities (Wadi et al., 2022).

Several participants within the current study also highlighted the importance of tailoring to account for previous weight management experience. No previous research has focused on the impact of cultural background or previous weight management attempts on programme effectiveness or experience within the NHS-DPP. However, previous research on the service user experiences of the pilot of the digital NHS-DPP reported similar findings to the current study, with a focus upon facilitating weight loss regardless of an individual’s starting weight (Ross et al., 2023). The service offered by the NHS-DPP has since expanded to allow for greater content tailoring for individual needs such as visual or hearing impairments and language support (called the ‘tailored remote service’) (NHS England, 2023). Thus, understanding service user experiences of this tailored version of the programme would provide a useful next step to understanding how this might improve service user experience and behaviour changes going forwards.

### Strengths and limitations

The current study forms part of a wider independent evaluation of the NHS-DPP involving four individual provider organisations delivering the national programme, investigating all stages of intervention development and delivery. Such independent evaluations of large-scale behaviour change programmes are rare. The current sample was composed of individuals who were observed taking part in the NHS-DPP as part of a previous programme of research (Hawkes et al., 2020b). Subsequently, the current study provides insight into service users’ experiences with the observed sessions, allowing for a richer evaluation of service delivery from both a service user and an external perspective.

The research team aimed to complete interviews with service users soon after they attended their final NHS-DPP session; however, in some cases, interviews took place up to five months after programme completion. The extended period between programme completion and conducting interviews in the current study introduced susceptibility to recall bias. The participants sometimes displayed difficulties in recalling information, particularly when asked to recall more specific experiences such as goal-setting activities. This may have affected individuals’ ability to recall exact experiences throughout the programme. However, the extended time period did allow the researchers to investigate the longer-term impacts of the NHS-DPP, such as behaviour change maintenance, and provide an insight into participants’ knowledge retention from a programme that is designed to produce long-term future benefits, which are often missing in programme evaluations. The fact that participants did display recall bias demonstrates the need for further maintenance support.

The majority of participants in the current study had engaged with more than 50% of the programme, consisting of individuals motivated to share their experiences of the NHS-DPP. However, other samples of users (e.g. those who were less engaged or who did not take up the NHS-DPP) would have different views, and this warrants further exploration in future research. However, in cases where this sample of engaged participants reported fewer positive experiences, it provides a strong argument for where refinements could be made to the future implementation of diabetes prevention programmes.

### Implications for practice

The current study has several implications for the implementation of diabetes prevention programmes. The findings of the current study highlighted the importance of accounting for individual differences in both intervention design and during referral. Future refinement of diabetes prevention programmes should consider ways of assessing individual needs and characteristics upon referral to ensure that participants are referred to an appropriate prevention programme such as a digital programme or culturally adapted programme to improve individual experiences and intervention success. Moreover, assessments could identify an individual’s prior knowledge of weight management, with more knowledgeable people grouped together in more discussion-based sessions to focus on overcoming individual barriers. Additionally, future programmes and versions of the NHS-DPP should be co-designed with different populations to ensure that they are culturally relevant and acceptable to different population groups.

Future intervention designs should aim to emphasise the importance of social support to facilitate behaviour change maintenance. Emphasis could be achieved through providing information concerning the impact of family on diet and behaviour change, the inclusion of examples of adopting behaviour changes with partners and family members, or inviting individuals to bring members of their social circle to sessions for them to learn how to support individuals with their diabetes prevention. Elsewhere, future intervention designs could be expanded to incorporate periodic follow-ups after programme completion to provide motivation to individuals to continue their behaviour changes or support individuals who have been unable to maintain their changes. Follow-ups may take the form of in-person meetings, follow-up telephone calls, or the provision of access to digital apps through which participants can access health monitoring tools and summaries of materials covered during the programme, particularly for programmes offering a form of digital intervention.

In recent years, there has been a substantial shift towards the online and virtual delivery of diabetes prevention programmes. Data from the current study are applicable to the in-person delivery of these programmes. However, evaluations of the digital NHS-DPP have also been conducted, including interviews with service users who took part in the digital service (Cheung et al., 2023; Moussas-Alvarez et al., 2025). Taken together, these qualitative insights from both modalities of delivery provide insights into shaping the implementation of large-scale diabetes prevention programmes.

### Implications for research

The current study identified a mindset shift towards healthier behaviours, even in the absence of behaviour change maintenance. Future research should aim to further test the presence of a mindset shift in individuals who have attended diabetes prevention programmes through more objective measures over a longer term. Moreover, the current study and previous research have focused on individuals who attended the NHS-DPP within the first two years after it was nationally implemented. The most recent rollout of the NHS-DPP now includes a ‘tailored remote service’ in addition to in-person and digital modalities, which provides the option for individuals attending the NHS-DPP to receive additional language and visual support in groups delivered remotely (NHS England, 2023). Future research should investigate whether the introduction of this tailored service improves individual experiences or outcomes.

## Conclusion

This study has highlighted that despite struggling to maintain behaviour changes, individuals who attend the NHS-DPP experience a shift in mindset toward healthier behaviours. Also, the participants highlighted how factors such as social support and individual circumstances, such as their starting weight, could impact on their experiences and subsequent behaviour changes. Increased cultural tailoring, particularly around dietary content, was identified as a key theme to increase engagement and behaviour change of participants. Future in-person diabetes prevention programmes could consider further individual tailoring and encouragement of social support to improve individual experiences during the programme and subsequent behaviour change maintenance.

## Data Availability

Transcripts of interviews analysed in the current study are not publicly available owing to confidentiality agreements with the provider organisations, as some information is commercially sensitive. Some datasets are available from the corresponding author upon reasonable request, although the authors will require the explicit permission of the relevant provider organisations. This study was performed in accordance with the Declaration of Helsinki and reviewed and approved by the North West Greater Manchester East NHS Research Ethics Committee (Reference: 17/NW/0426, 2nd August 2017).

## Appendices

### Appendix A. Standards for Reporting Qualitative Research (SRQR)

O’Brien B.C., Harris, I.B., Beckman, T.J., Reed, D.A., & Cook, D.A. (2014). Standards for reporting qualitative research: a synthesis of recommendations. *Academic Medicine, 89(9)*, 1245-1251.

**Table A1.**

No. Topic	Item	Page no.
**Title and abstract**		
S1 Title	Concise description of the nature and topic of the study identifying the study as qualitative or indicating the approach (e.g. ethnography, grounded theory) or data collection methods (e.g. interview, focus group) is recommended	1 (Title Page)
S2 Abstract	Summary of key elements of the study using the abstract format of the intended publication; typically includes objective, methods, results, and conclusions	2–3
**Introduction**		
S3 Problem formulation	Description and significance of the problem/phenomenon studied; review of relevant theory and empirical work; problem statement	4–7
S4 Purpose or research question	Purpose of the study and specific objectives or questions	6–7
**Methods**		
S5 Qualitative approach and research paradigm	Qualitative approach (e.g. ethnography, grounded theory, case study, phenomenology, narrative research) and guiding theory if appropriate; identifying the research paradigm (e.g. positivist, constructivist/interpretivist) is also recommended	7 and 9–10
S6 Researcher characteristics and reflexivity	Researchers’ characteristics that may influence the research, including personal attributes, qualifications/experience, relationship with participants, assumptions, or presuppositions; potential or actual interaction between researchers’ characteristics and the research questions, approach, methods, results, or transferability	7–9 and 10–11
S7 Context	Setting/site and salient contextual factors; rationale^a^	7–9 and 10–11
S8 Sampling strategy	How and why research participants, documents, or events were selected; criteria for deciding when no further sampling was necessary (e.g. sampling saturation); rationale^a^	7–8
S9 Ethical issues pertaining to human subjects	Documentation of approval by an appropriate ethics review board and participant consent, or explanation for lack thereof; other confidentiality and data security issues	11 and Declarations statements
S10 Data collection methods	Types of data collected; details of data collection procedures including (as appropriate) start and stop dates of data collection and analysis, iterative process, triangulation of sources/methods, and modification of procedures in response to evolving study findings; rationale^a^	7–9
S11 Data collection instruments and technologies	Description of instruments (e.g. interview guides, questionnaires) and devices (e.g. audio recorders) used for data collection; if/how the instrument(s) changed over the course of the study	8–9 and Appendix B
S12 Units of study	Number and relevant characteristics of participants, documents, or events included in the study; level of participation (could be reported in results)	11 and Table 2
S13 Data processing	Methods for processing data prior to and during analysis, including transcription, data entry, data management and security, verification of data integrity, data coding, and anonymization/deidentification of excerpts	8–10
S14 Data analysis	Process by which inferences, themes, etc., were identified and developed, including researchers involved in data analysis; usually references a specific paradigm or approach; rationale^a^	9–10 and Appendix C
S15 Techniques to enhance trustworthiness	Techniques to enhance trustworthiness and credibility of data analysis (e.g. member checking, audit trail, triangulation); rationale^a^	9–10
**Results/Findings**		
S16 Synthesis and interpretation	Main findings (e.g. interpretations, inferences, and themes); might include development of a theory or model, or integration with prior research or theory	11–24
S17 Links to empirical data	Evidence (e.g. quotes, field notes, text excerpts, photographs) to substantiate analytic findings	13–24
**Discussion**		
S18 Integration with prior work, implications, transferability, and contribution(s) to the field	Short summary of main findings; explanation of how findings and conclusions connect to, support, elaborate on, or challenge conclusions of earlier scholarship; discussion of scope of application/generalizability; identification of unique contribution(s) to scholarship in a discipline or field	24–30
S19 Limitations	Trustworthiness and limitations of findings	27–28
**Other**		
S20 Conflicts of interest	Potential sources of influence or perceived influence on study conduct and conclusions; how these were managed	Declarations and Funding statements
S21 Funding	Sources of funding and other support; role of funders in data collection, interpretation, and reporting	Funding and Acknowledgements statements

^a^ The rationale should briefly discuss the justification for choosing that theory, approach, method, or technique rather than other options available, the assumptions and limitations implicit in those choices, and how those choices influence study conclusions and transferability. As appropriate, the rationale for several items might be discussed together.

### Appendix B. Full Interview Schedule

**Evaluating the NHS Diabetes Prevention Programme**

**Question schedule**

Thank you for agreeing to take part in this interview. Please let me know if you would like to take a break or pause the recording at any point, or if you would like to stop the interview. Please note that the information we collect will be kept securely and confidentially in accordance with data protection laws, as described on the patient information sheet. The interview should take no more than one hour.

**Questions about the ‘Healthier You’ Diabetes Prevention Programme**

1. Before the ‘Healthier You’ sessions started, what did you expect from the course?

- Was the course similar or different to what you were expecting?

2. What did you think of the programme, now that you’ve been on it?

-What did you like about it? What did you not like? What was important for you? How could it be improved?

3. How useful did you find the course?

- What is the main thing in your life that changed for you as a result of the course?

4. What do you think the main aim of the course is?

- How does it try to do that?

- Was this made clear to you at the start of the course?

5. What do you think of the information given on the course about diabetes and preventing diabetes?

- Was it the right type, amount, format, level of difficulty?

- What was the most important thing you learned in the course?

6. As part of the course, did the health coach ask you to set any goals or targets for your physical activity, diet, or weight loss?

- How did you get on with that? (Choosing, setting, working towards a goal?)

- How *useful* did you find it for helping you change your physical activity or diet?

- How do you think goals *work* to help people change their physical activity or diet?

7. Did the health coach ever ask you to make a more detailed plan for changing your diet or physical activity, sometimes called an action plan, a plan for change, or a ‘what am I going to do now’ sheet?

-- How did you get on with those? (Making a plan, sticking to it?)

- How *useful* did you find them for helping you change your physical activity or diet?

- How do you think action plans *work* to help people change their physical activity or diet?

8. Were you asked at any point to keep track of your physical activity or diet or weight, by using a daily diary or a step counter, or a logbook?

- How did you get on doing that?

- How *useful* did you find it for helping you change your physical activity or diet?

-- How do you think logbooks *work* to help people change their physical activity or diet?

9. Did the health coach ask you to do any problem solving, where you look at barriers getting in the way of making healthy changes and come up with solutions for overcoming the barriers?

- How did you get on doing that? How often were you able to do problem solving or use any of the suggested solutions after the class?

- How *useful* did you find that for helping you change your diet or activity?

- How do you think problem solving *works* to help people change their diet and activity?

10. Did the health coach give you any feedback on changes to your weight, blood glucose levels, waist circumference, diet or physical activity?

- How useful did you find it do have that feedback? How easy was it to understand?

- How did you feel being given that feedback?

- How timely was the feedback?

11. During your time in the course, how important were your family and friends in sticking to the programme and making changes to your lifestyle?

- What impact did their views and behaviours have?

12. Is there anything else about the course we haven’t talked about yet that you’d like to tell me about or report back on?

Thank you very much for taking the time to take part in our research.

### Appendix C. Full Analytical Framework

**General Experiences**

**Table:**

Code (Description)			
Journey before NHS DPP Previous weight management attempts, how they discovered the programme, referral process, accessing the group	Experiences (other) *Experiences about the programme not captured by other codes*	Intervention Effectiveness *Changes in weight and/or blood sugar levels during programme*	Journey after NHS DPP *Engagement with subsequent programmes, weight/blood sugar levels, habits after programme*
***Sub Codes***			
Individual circumstances *Comments about individual’s starting weight and circumstances such as health conditions and how it influenced their experiences*		Behaviour Change *Specific behaviour changes, habit formation/changes (or lack of changes) i.e. dietary swaps, reduced alcohol consumption, increase physical activity* Behaviour Change maintenance *Comments about how long behaviour changes were maintained by the individual and group members—includes discussion regarding use of BCTs* Expectations *Comments about whether the programme met their initial expectations about the specific programme or general weight management programmes*	

**Organisational Experiences**

**Table:**

Code (Description)				
Course Structure Comments regarding course duration, number of sessions, number of follow ups	Positive comments of Session Organisation *Group size, organisation of sessions (e.g. cancelled/rearranged sessions), notice period, justification for comment*	Negative Comments of Session Organisation *Group size, organisation of sessions (e.g. cancelled/rearranged sessions), notice period, justification for comment*	Venue *Comments about intervention venue, appropriateness, location etc.*	Health check ins *Comments about experiences relating to health check ins i.e. weigh ins/glucose monitoring*
***Sub Codes***				
		Suggested Improvements *Comments regarding how they felt Session Organisation could be improved*		Feedback *Feedback/lack of feedback from facilitator*

**Educational Experiences (Includes practical activities and group discussions)**

**Table:**

Code (Description)				
Content Covered *Topics covered, key messages, weight loss, sugar control*	Positive comments of Educational Content *Topic, delivery, reason for comment*	Negative Comments of Educational Content *Topic, delivery, reason for comment*	Content Complexity *Level of content provided, was it appropriate, justification for reason*	Content Tailoring *Adaptation/lack of adaptation for different backgrounds and needs i.e. ethnicity, SES, dietary habits*
***Sub Codes***				
Mode of Delivery *How content was delivered i.e. practical examples, verbal explanations. Modes of delivery participants liked/disliked.*		Suggested Improvements *Comments regarding how they felt Educational Content could be improved*	Understanding *Level of participant understanding of content delivered, ability to recall content*	Personalisation *Adaptation/lack of adaptation to individual experiences i.e. knowledge level, personal goals*

**Support Experiences**

**Table:**

Code (Description)				
Comments regarding the Facilitator *Connection with facilitator, facilitator feedback, how facilitator helped them/didn’t help them, changes in facilitator*	Positive experiences of Social Support *Family and/or friends providing practical and/or emotional support etc.*	Negative Experiences of Social Support *Family and/or friends being discouraging and/or unsupportive etc.*	Group Dynamics *Experiences with fellow group members, practical talks, group characteristics, how groups impacted experience*	
***Sub Codes***				
	Social Outings *Experiences at specific social outings i.e. meals out, weddings, birthdays*	Social Outings *Experiences at specific social outings i.e. meals out, weddings, birthdays* Suggested Improvements *Comments regarding how they felt Social support could be improved*		

**Materials experiences**

**Table:**

Code (Description)				
Materials Provision *Comments about provision of additional materials i.e. workbooks, handouts, technology*	Positive comments of Additional Materials *Structure, duration, delivery, reason for comment*	Negative Comments of Additional Materials *Structure, duration, delivery, reason for comment*	Step Counters *Comments regarding the provision and effectiveness of step counters*	Use of materials outside sessions *How participants engaged with materials outside of organised sessions i.e. homework, food diaries*
***Sub Codes***				
		Suggested Improvements *Comments regarding how they felt Additional Materials could be improved*		
